# Acoustic Trauma Increases Cochlear and Hair Cell Uptake of Gentamicin

**DOI:** 10.1371/journal.pone.0019130

**Published:** 2011-04-28

**Authors:** Hongzhe Li, Qi Wang, Peter S. Steyger

**Affiliations:** Department of Otolaryngology/Head and Neck Surgery, Oregon Hearing Research Center, Oregon Health and Science University, Portland, Oregon, United States of America; Dalhousie University, Canada

## Abstract

**Background:**

Exposure to intense sound or high doses of aminoglycoside antibiotics can increase hearing thresholds, induce cochlear dysfunction, disrupt hair cell morphology and promote hair cell death, leading to permanent hearing loss. When the two insults are combined, synergistic ototoxicity occurs, exacerbating cochlear vulnerability to sound exposure. The underlying mechanism of this synergism remains unknown. In this study, we tested the hypothesis that sound exposure enhances the intra-cochlear trafficking of aminoglycosides, such as gentamicin, leading to increased hair cell uptake of aminoglycosides and subsequent ototoxicity.

**Methods:**

Juvenile C57Bl/6 mice were exposed to moderate or intense sound levels, while fluorescently-conjugated or native gentamicin was administered concurrently or following sound exposure. Drug uptake was then examined in cochlear tissues by confocal microscopy.

**Results:**

Prolonged sound exposure that induced temporary threshold shifts increased gentamicin uptake by cochlear hair cells, and increased gentamicin permeation across the strial blood-labyrinth barrier. Enhanced intra-cochlear trafficking and hair cell uptake of gentamicin also occurred when prolonged sound, and subsequent aminoglycoside exposure were temporally separated, confirming previous observations. Acute, concurrent sound exposure did not increase cochlear uptake of aminoglycosides.

**Conclusions:**

Prolonged, moderate sound exposures enhanced intra-cochlear aminoglycoside trafficking into the stria vascularis and hair cells. Changes in strial and/or hair cell physiology and integrity due to acoustic overstimulation could increase hair cell uptake of gentamicin, and may represent one mechanism of synergistic ototoxicity.

## Introduction

Aminoglycosides are critical for treating life-threatening Gram-negative bacterial infections, e.g., bacterial sepsis and meningitis [1], [2]. Yet, aminoglycosides also induce cytotoxicity in the kidney and cochlea. After systemic administration, inner ear sensory hair cells and kidney proximal tubule cells preferentially retain aminoglycosides [3], display greater pharmacological sensitivity and cytotoxicity to these drugs than other cell types in the body [review by Rybak et al.[4]].

Acoustic trauma occurs in many environments, including occupational (e.g., industrial, military), or recreational (e.g., musical concerts, hunting) settings. Sound trauma-induced cochlear pathologies include increased endocytosis, vacuolation, mitochondrial lesions, elevation of intracellular Ca^2+^ concentrations and the generation of reactive oxygen species that can lead to hair cell death and permanent hearing loss [5], [6], [7]. Moderate acoustic damage can also be reversible, as temporary threshold shifts (TTS), due to acute exposure to high sound levels or chronic exposure to intermediate sound levels that induce reversible physiological changes in hair cells and the blood-labyrinth barrier [8], [9], [10].

Combining acoustic and ototoxic insults leads to synergistic ototoxicity, that is, potentiation of aminoglycoside-induced ototoxicity by acoustic trauma. This was first observed in the 1960s when animals receiving aminoglycosides appeared to be more susceptible to noise-induced hearing loss [11] and confirmed by subsequent studies [12], [13], [14], [15]. The same ototoxic mechanism is likely responsible for the increased deafness risk in pre-term infants from neonatal intensive care units [16], [17] and in wounded soldiers [18], [19], [20], [21], [22]. Multiple cochlear events may be responsible for sound-and-drug induced synergy. Aminoglycosides slowly permeate through the mechanoelectrical transduction (MET) channel on the apical surface of hair cells, blocking the rapid depolarizing transduction current of MET channels, preventing functional ion channel kinetics [23], [24]. An increase in the open probability of, and current through, MET channels during sound exposure could increase aminoglycoside uptake, and form one underlying mechanism for sound and drug synergy [17]. Alternatively, pathological modifications, including variation in vascular permeability [25], in the cochlear lateral wall are induced by acoustic overstimulation. These modifications may increase aminoglycoside permeation into endolymph that bathes the apical surfaces of hair cells. Trans-strial trafficking of systemic aminoglycosides into endolymph is considered to be a primary route for aminoglycoside loading of sensory hair cells [26]. In this study, we hypothesized that sound exposure enhances the intra-cochlear trafficking of aminoglycosides, and consequently hair cell uptake of aminoglycosides.

To test the hypothesis, mice were exposed to moderate or intense sound levels either concurrently or prior to gentamicin treatment. Fluorescently-conjugated (GTTR) or native gentamicin was administered during or after a variety of sound exposures. Here, we report that (i) chronic acoustic overstimulation increased hair cell uptake of systemic aminoglycosides; (ii) acute, concurrent sound and systemic aminoglycoside exposure did not increase hair cell uptake of aminoglycosides; and (iii) broader examination of cochlear tissues revealed that marginal cells in the stria vascularis exhibit increased gentamicin uptake following chronic, sound-induced TTS, representing one potential mechanism for sound-then-drug induced ototoxic synergy.

## Results

### Prolonged prior sound exposure enhanced gentamicin uptake in hair cells

**Figure 1:**
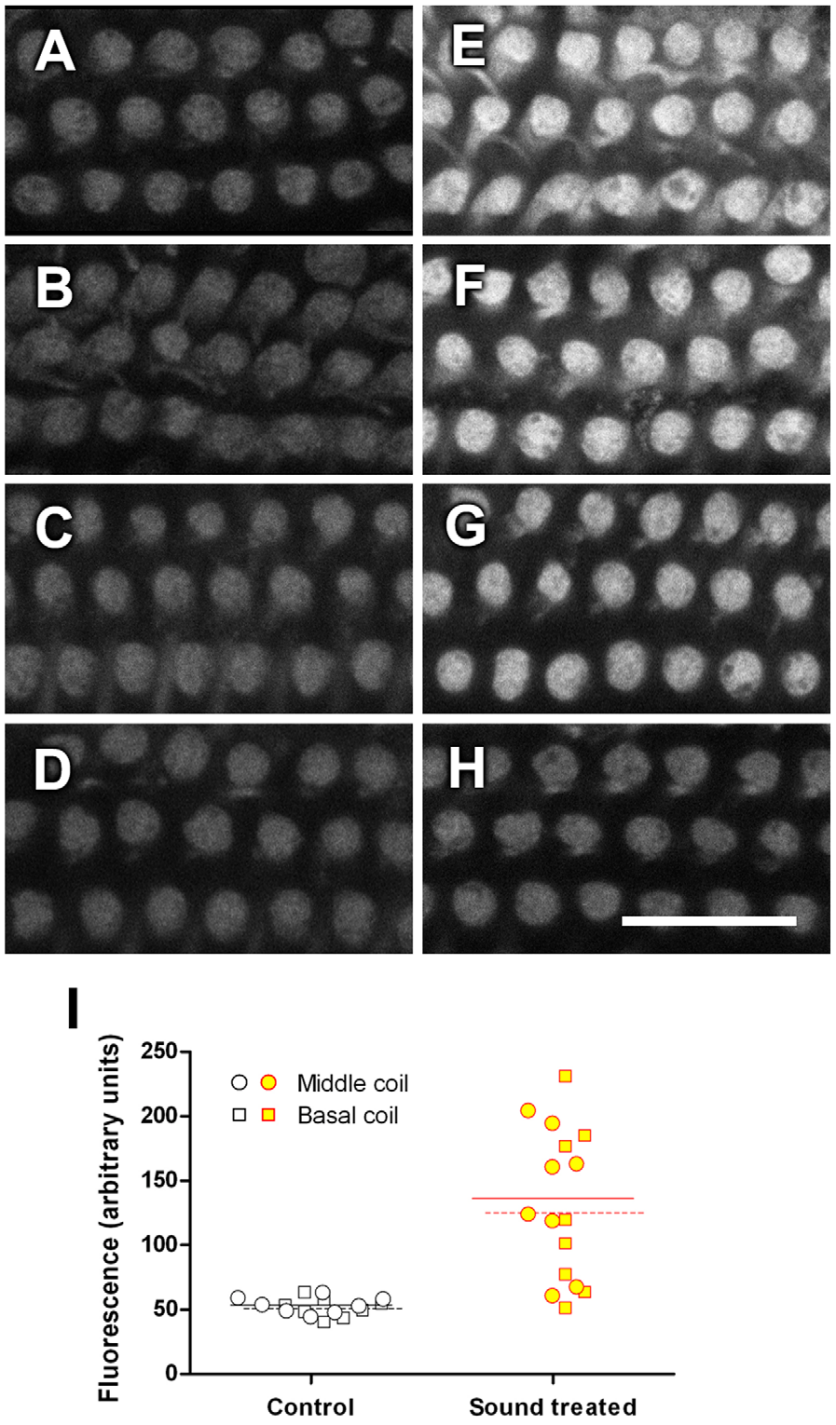
Prior prolonged sound exposure increased GTTR uptake in OHCs. GTTR (2 mg/kg) was injected i.p. for 30 minutes after an 18-hour WBN exposure over 3 days (6 hours per day, 86 dB SPL). Increased GTTR fluorescence intensities were observed in OHCs from the middle coil of cochleae from the majority of individual animals (**E–H**), compared to control (non-sound exposed) animals (**A–D**). Scale bar in H is 20 µm. **I**: The fluorescence intensity of the cuticular plate region of each OHC was scored from each confocal stack. Four stacks were imaged from each animal. Two sites were from the middle coil (circle) and 2 sites were from the basal coil (square). OHCs from 3 of 4 examined animals exhibited robust increase in GTTR fluorescence after prolonged prior sound exposure, compared to control OHCs (n = 4). Horizontal lines depict the group mean of fluorescence intensity. Solid line: middle coil; dashed line: basal coil. The Mann-Whitney nonparametric test indicated that prolonged sound exposure exerted a statistically significant effect on GTTR fluorescence in OHC cuticular plates in both middle coil (p<0.001) and basal coil (p<0.01) locations compared to control cuticular plates.

In this study, to determine if prolonged wide-band noise (WBN) exposure increased gentamicin uptake, mice were exposed to WBN for 18 hours (86 dB SPL, over 3 days, 6 h per day). A moderate sound level was intentionally selected to mimic the environment in neonatal intensive care units [16]. After sound exposure on the 3^rd^ day, mice were allowed to rest for 30 minutes prior to i.p. injection with GTTR (2 mg/kg). Mice were then kept in quiet for another 30 minutes prior to cochlear tissue collection. In 3 out of 4 sound-exposed mice (Fig. 1E–H), robust GTTR fluorescence was observed in OHCs in both middle and basal regions of the cochlea, compared to weaker GTTR fluorescence in non-sound-exposed control mice (Fig. 1A–D). GTTR uptake in some sound-exposed OHCs was very robust that some pixels were saturated in intensity, i.e., the fluorescence intensity in these pixels was likely underestimated. The increase of fluorescence intensities was statistically significant (Fig. 1I and Fig. 3F). Thus, prolonged prior sound exposure greatly enhanced GTTR uptake in OHCs, although with some inter-animal variation (summarized in Fig. 1I). Similar results were observed when GTTR treatment was overlapped by the final 30-min sound exposure on day 3 (Figure S1).

**Figure 2:**
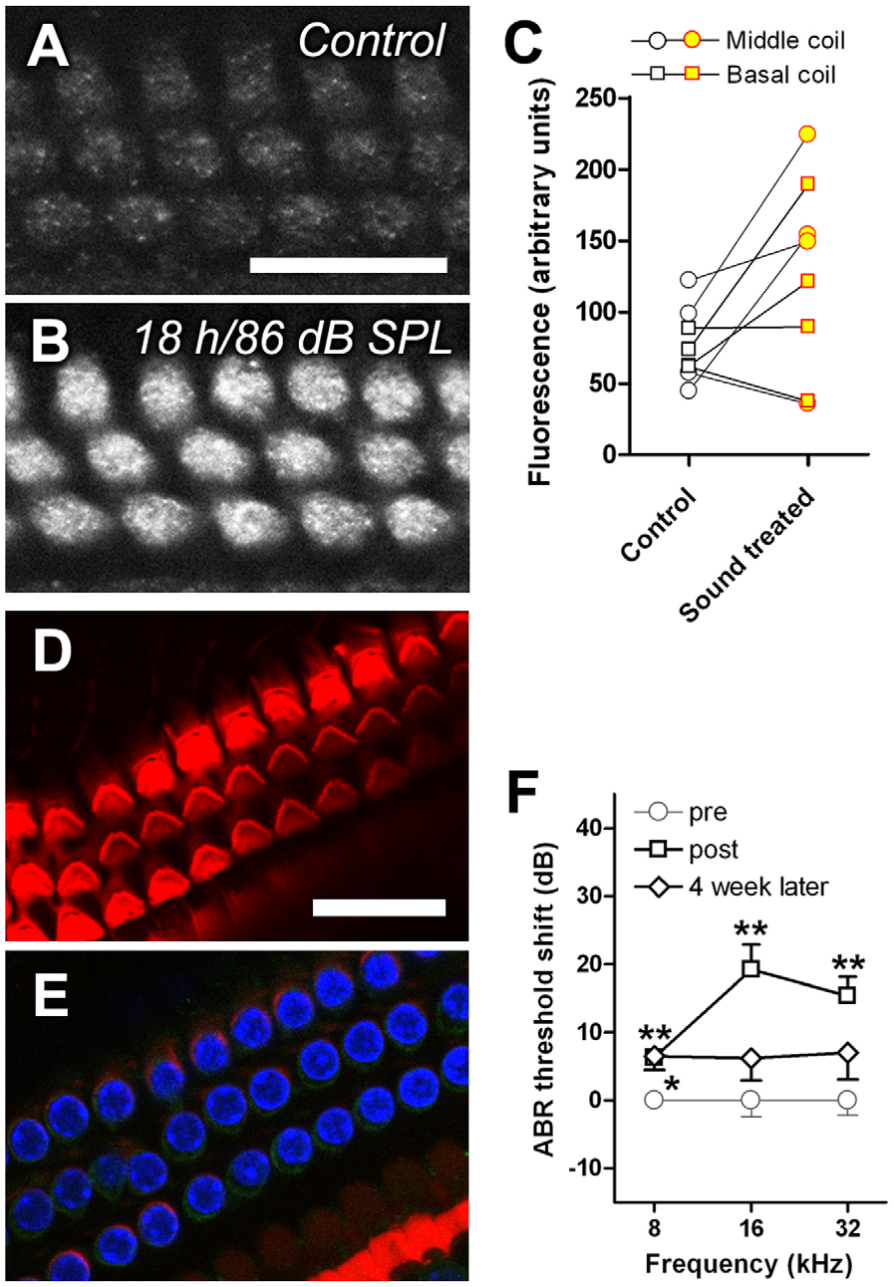
Prolonged sound exposure increased gentamicin uptake in OHCs. Gentamicin (200 mg/kg) was injected i.p. for 30 minutes following an 18-hour WBN exposure over 3 days (6 hours per day, 86 dB SPL). Increased gentamicin immunofluorescence was generally observed in basal sound-exposed OHCs (**B**) compared to control (non-sound exposed) animals (**A**). Scale bar in A = 20 µm. **C**: The immunofluorescence intensity of OHC cuticular plates from either middle coils (circle) or basal coils (square) were increased in 3 of 4 animals after chronic moderate sound exposure (p<0.05, paired t-test). Immunoprocessing for each batch of animals were carried out simultaneously for each pair-wise comparison. Chronic moderate sound exposure did not disrupt hair bundle morphology determined by phalloidin labeling (**D**, *red*), nor nuclei integrity by DAPI staining (**E**, *blue*); *green*, anti-gentamicin IgG. Scale bar  = 20 µm. **F**: This sound exposure paradigm produced a temporary elevation in hearing thresholds (n = 6) that largely recovered within 4 weeks. Error bar is s.e.m. *p<0.05, **p<0.01.

**Figure 3:**
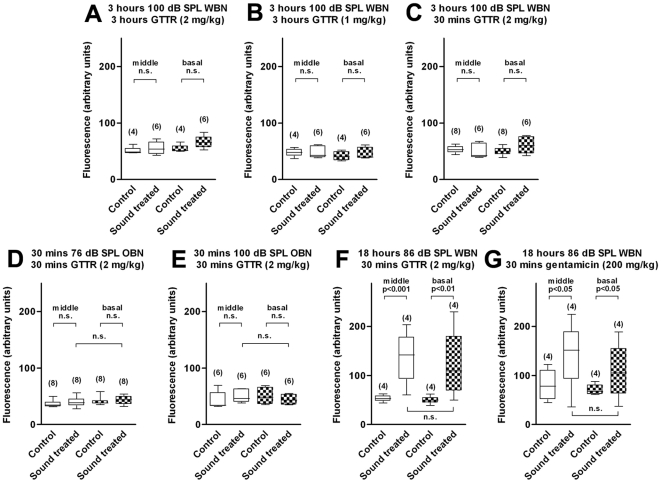
Concurrent sound exposure did not increase GTTR uptake in OHCs. The intensity of GTTR fluorescence in OHC cuticular plates was scored from either middle or basal coils, and data are in box and whisker plots, which respectively represent the lower and upper quartiles, and the minimum and maximum of each data set. **A**: WBN (100 dB SPL) and GTTR (2 mg/kg, i.p.) simultaneously administered for 3 hours. **B**: WBN (100 dB SPL) and GTTR (1 mg/kg, i.p.) co-administered for 3 hours. **C**: GTTR (2 mg/kg, i.p.) administered 30 minutes prior to the end of 3 hour WBN (100 dB SPL) exposure. Sound was delivered in open field in A–C, and GTTR fluorescence obtained from the left cochlea only. **D**: OBN of 76 dB SPL (centered at 12 kHz) and GTTR (2 mg/kg, i.p.) co-administered for 30 minutes. **E**: OBN of 100 dB SPL (centered at 12 kHz) and GTTR (2 mg/kg, i.p.) co-administered for 30 minutes. Sound was delivered in a closed tube system in D and E, while GTTR fluorescence intensities were compared between sound-exposed left ears and non-sound exposed right ears. **F**: GTTR (2 mg/kg) was injected i.p. for 30 minutes after an 18-hour 86-dB-SPL WBN exposure over 3 days (same dataset as Fig. 1I). **G**: Gentamicin (200 mg/kg) was injected i.p. for 30 minutes following an 18-hour 86-dB-SPL WBN exposure over 3 days (same dataset as Fig. 2C). n.s. =  not significant, by either unpaired (A, B, C and F) or paired (D, E and G) t-test. Experiments were not compared across conditions/figure panels.

We repeated this experiment using prolonged sound exposure and native gentamicin, followed by immunofluorescence. The monoclonal gentamicin antisera used in this study produced a monotonic increase in fluorescence intensity with increasing gentamicin dose (Figure S2). Gentamicin (200 mg/kg i.p.) was given to mice for 30-min following prolonged sound exposure (18 hour WBN at 86 dB SPL over 3 days, 6 h per day). Control animals were exposed to gentamicin only for 30 min. In 3 out of 4 sound-exposed mice, OHCs displayed greater gentamicin immunofluorescence compared to non-sound-exposed mice (Fig. 2A–C and Fig. 3G). Neither hair bundle damage, nor nuclear abnormalities, were detectable in phalloidin (Fig. 2D) and DAPI-stained (Fig. 2E) whole mounts from prolonged sound-exposed cochleae. This paradigm of sound exposure induced significant TTS at 8, 16 and 32 kHz, and substantial recovery 4 weeks later (Fig. 2F). Together with earlier experiments, these data indicate that chronic, moderate prior sound exposure can increase hair cell uptake of gentamicin.

To investigate whether sound stimulation itself increased drug uptake by hair cells, we presented WBN (100 dB SPL) for a shorter 3 hour period in an open field, and concurrently administered GTTR (i.p.) systemically. Concurrent sound and GTTR exposure for 3 hours did not enhance OHC uptake of GTTR in middle or basal cochlear coils (Fig. 3A). To rule out the possibility that GTTR trafficking mechanisms were saturated between the strial vasculature and the organ of Corti, additional experiments were done by reducing the GTTR level to 1 mg/kg (Fig. 3B), or reducing the GTTR (2 mg/kg) exposure period to 30 minutes (Fig. 3C). Neither exposure paradigm altered the degree of GTTR uptake by sound-exposed OHCs compared to controls.

We then used closed tube sound delivery of octave-wide band noise (OBN), and to provide frequency-specific sound exposure. The OBN was centered at 12 kHz, corresponding to a cochlear location of 33% from the apex [27], approximately the middle coil region of the cochlea, and OHCs were imaged. A 30-minute concurrent sound and GTTR exposure did not enhance GTTR uptake by OHCs, at either moderate (76 dB SPL, Fig. 3D) or intense (100 dB SPL, Fig. 3E) sound levels over control mice. In addition, a frequency-specific enhancement of GTTR uptake by OHCs was not observed, i.e., similar fluorescence levels occurred in OHCs from the middle coil and basal coil in sound-exposed ears. These experiments were repeated using gentamicin (200 mg/kg i.p.), and no differential gentamicin immunofluorescence was observed between sound-exposed and control ears, nor between middle and basal coils (data not shown).

### Heterogeneous enhancement of gentamicin uptake by strial cells

**Figure 4:**
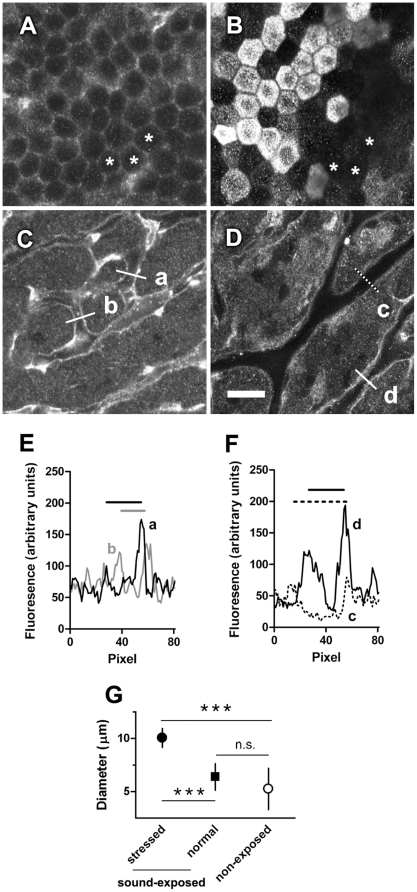
Prolonged, moderate sound exposure increases gentamicin uptake in a subpopulation of cells in the stria vascularis. In non-sound-exposed strial tissues (A, C and E), moderate gentamicin immunofluorescence was observed in marginal cells (**A**, asterisks), with more intense fluorescence in the strial vasculature (**C**). After prolonged sound exposure (18 hours over 3 days, WBN 86 dB SPL; B, D and F), a subpopulation of marginal cells showed increased gentamicin immunofluorescence compared to adjacent marginal cells (**B**, asterisks, normal region) and non-sound-exposed marginal cells (A). **D**: The strial vasculature below the subpopulation of intensely-labeled marginal cells displayed reduced gentamicin immunofluorescence (**c**) compared to strial capillaries below marginal cells with baseline levels of gentamicin immunolabeling (**d**), or non-sound-exposed strial capillaries (C). Scale bar  = 20 µm, fits all image panels. **E**: Plot profiles of lines **a** and **b** delineated in (C), showing the regular diameters of blood vessels without sound exposure. **F**: Plot profiles of lines **c** and **d** delineated in (D), showing representative vasodilation and reduced endothelial fluorescence in capillaries below the intensely labeled marginal cells following sound exposure. Bars above fluorescent traces depict the diameter of capillary of interest. **G**: Sound-induced vasodilation in capillaries below intensely labeled marginal cells, but not in adjacent regions with marginal cells with baseline levels of gentamicin immunofluorescence (***p<0.001). Error bar is s.d. n.s. =  not significant.

Typically, the most intense gentamicin immunofluorescence in control (non-sound-exposed) strial tissues was observed in the strial vasculature (Fig. 4C), as for GTTR [26], [28]. When we examined the stria vascularis after chronic moderate sound exposure (same animals as in Fig. 2), a subpopulation of marginal cells exhibited more intense gentamicin immunofluorescence (Fig. 4B) compared to adjacent marginal cells or marginal cells from control (non-sound-exposed) mice (Fig. 4A). In addition, the fluorescent intensity of the vasculature in sound-exposed stria vasculari (Fig. 4D) was reduced compared to control (non-sound-exposed) strial tissues (Fig. 4C); and the most intense fluorescence in sound-exposed strial vasculari was localized in individual groups of marginal cells (Fig. 4B). Furthermore, chronic sound exposure also increased the diameter of strial blood vessels (*i.e.* vasodilation) in the same strial regions with intensely-labeled marginal cells compared to adjacent strial regions without intensely-labeled marginal cells or in strial tissues from non-sound-exposed animals (Fig. 4E, F and G).

**Figure 5:**
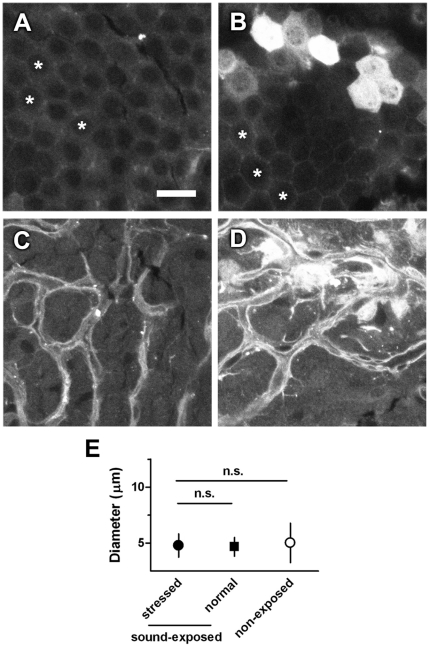
Intense sound exposure, followed by 18 hours recovery also increases GTTR uptake in the stria vascularis. In non-sound exposed strial tissues (A and C), moderate GTTR fluorescence was observed in marginal cells (**A**, asterisks) and intra-strial cells (**C**), with most intense GTTR fluorescence in the strial vasculature. Scale bar  = 20 µm, fits all image panels. In intense-sound exposed tissues (6 hours, OBN 100 dB SPL, 18-hour recovery; **B** and **D**), a subset of marginal cells show increased GTTR fluorescence compared to adjacent marginal cells (B, asterisks), and marginal cells from non-sound exposed cochleae (A, asterisks). Directly below the intensely fluorescent marginal cells in (B), cells in the intra-strial layer also exhibited increased GTTR fluorescence. **E**: After 18 hours of recovery, no vasodilation (increase in vessel diameter) was observed, in the strial vasculature from either sound or non-sound-exposed cochleae (stressed: blood vessels from strial regions with enhanced uptake of GTTR in sound exposed cochleae; normal, blood vessels from strial regions with normal uptake of GTTR in sound-exposed cochleae). Error bar is s.d.

Capillary or blood vessel diameter is highly correlated to strial blood flow, an attribute that can be consistently monitored *in vivo*, and can vary during sound exposure [29]. Noise-induced variation in blood flow within the strial vasculature is transient, and eventually recovers to pre-exposure values [30] within hours. Mice were exposed to 100 dB SPL octave-wide band noise (8–16 kHz) for 6 hours. The next morning, to allow recovery of any transient diameter changes in strial vessels, mice were injected with GTTR (2 mg/kg i.p.), and cochlear tissues collected 30 minutes later. In these mice, increased GTTR fluorescence was observed in individual groups of marginal cells (Fig. 5B), compared to non-sound-exposed control strial cells (Fig. 5A). Directly below these intensely fluorescent marginal cells, the intra-strial tissues also showed a corresponding increase in GTTR fluorescence (Fig. 5D), without evidence of vasodilation (Fig. 5E), nor a decrease in fluorescence in the strial vasculature (endothelial cells). These regions of increased drug uptake in sound-exposed tissue were observed in 3 of 8 sound-exposed cochleae, using GTTR or gentamicin (immuno)fluorescence. A retrospective examination of cochleae in Fig. 1, also revealed sound-induced heterogeneous uptake of GTTR by strial cells in cochleae that displayed robust GTTR uptake in OHCs. This suggests that chronic sound exposure or sound-induced trauma can enhance aminoglycoside trafficking into the stria vascularis, as well as hair cells, hours after cessation of exposure to loud sounds.

## Discussion

Preventing the ototoxic synergy of noise and aminoglycosides can be best achieved by using non-ototoxic bactericidal drugs, and by attenuating perceived sound levels when life-saving aminoglycoside therapy is required. However, this is not always possible, e.g., prior exposure to blasts and explosions followed by wound treatment with aminoglycosides in war zones [18], [19], [20], [21], [22]; or chronic exposure to moderate sound levels and aminoglycosides by pre-term infants in intensive care units [16]. Therefore, it is critical to understand how sound exposure can enhance the unwanted ototoxic side-effects of the clinically-indispensable aminoglycosides.

Previous animal studies of sound and aminoglycoside synergy measured hearing sensitivity and constructed cytocochleograms of hair cell survival [13], [14]; they did not measure drug uptake *in vivo*. Gentamicin is present within OHCs before onset of drug-induced ototoxicity [31], emphasizing the need to identify and prevent the underlying mechanism of drug trafficking. Since prior sound exposures that induce TTS (or greater pathologies) can increase hair cell uptake of gentamicin *in vivo*, interventions that inhibit trafficking of aminoglycosides into the cochlea likely represent one potential clinical strategy to prevent sound-then-drug ototoxic synergism.

Previous studies of simultaneous sound and drug exposure took place over many days, and are different from the acute concurrent experimental paradigms used here. In a study by Collins [32] showed that if sound exposure was given on the first day of a 10-day gentamicin course there was considerable loss of hair cells; if given on the 10^th^ day the loss was several times less. Other studies report ototoxic synergy when aminoglycoside treatment followed acoustic overstimulation, but not when sound exposure followed drug treatment [see review by Li and Steyger [17]]. We believe that any weak synergistic effect observed in a drug-then-sound paradigm, as by Hayashida and colleagues [33], is due to a compound effect between residual aminoglycoside-induced damage and subsequent sound exposure.

Despite attempts to understand the impact of noise on permeability variation of blood-labyrinth barrier [such as [10]], moderate sound levels that more relevant to clinical situations were rarely tested. In this study, we demonstrate that chronic or damaging sound exposures at moderate levels can enhance cochlear uptake of gentamicin *in vivo*, which has not been previously shown (to our knowledge). GTTR enters hair cells within 30 minutes after systemic administration without sound stimulation [26]. Exogenous stimulation such as administration of vasoactive peptides, e.g., histamine, can significantly reduce OHC uptake of GTTR within 30 minutes [34]. Thus, the 30 minute timepoint represents a good baseline of GTTR uptake with sufficient dynamic range to observe any sound-induced modulation of OHC uptake of GTTR fluorescence.

### Aminoglycoside uptake by hair cells

We observed that chronic moderate sound exposure can increase hair cell uptake of GTTR or gentamicin. Variance of OHC fluorescent intensity in control (non-sound exposed) cochleae with 30 minute systemic GTTR exposure was lower than in gentamicin-immunolabeled OHCs (compare Fig. 1I with Fig. 2C). The coefficient of variation of control mice in Fig. 1 was 0.13, and 0.33 for control mice in Fig. 2 (gentamicin immunofluorescence). The difference in variance is statistically significant (f-test, p<0.0001) if the distributions are assumed normal. This difference is likely a result of the intrinsic variability inherent in immunoprocessing tissues through two stages of antigen-antibody reaction. Nonetheless, chronic sound exposure robustly increased the fluorescence signal by either method. However, GTTR is preferable for differential testing of individual experimental variables over gentamicin immunolabeling due to its consistent dose-intensity relationships compared to immunofluorescence (see Figure S2) [26].

Aminoglycosides severely attenuate the permeation kinetics of K^+^ and Ca^2+^ through MET channels while simultaneously slowly permeating through the channel itself [23], [24]. Several studies have reported that dihydrostreptomycin and GTTR enter hair cells through MET channels [24], [26], [35]. Our acute experimental data show that sound exposure does not directly increase OHC uptake of aminoglycosides through the MET channels *in vivo*, which fits with the hypothesis that the open probability of OHC MET channels remains relatively constant, whether at rest or during stimulation [36].

Prolonged, and/or intense sound exposure that induces TTS increased OHC uptake of gentamicin *in vivo*. One hypothesized mechanism for TTS is the breaking of stereociliary tip-links that gate the MET channels [37]. If this occurs *in vivo*, then the decoupled MET channels are unlikely to be responsible for increased OHC uptake of gentamicin during TTS. This suggests that sound trauma-enhanced uptake of aminoglycosides by OHCs occurs by a different mechanism, activated by prolonged sound exposure, and/or other pathologies induced in hair cells or the stria vascularis.

### Strial blood-labyrinth barrier and gentamicin trafficking

Sound-induced damage in the cochlea is not limited to the organ of Corti; reversible pathological changes also occur in the stria vascularis, including modulation of cochlear blood flow [29], [30], induction of micro-ischemia, and changes in vascular permeability [10], [25]. The basal region of sound-exposed cochleae frequently exhibited changes in vessel diameter and increased loading of GTTR or gentamicin in a subset of marginal cells, after chronic moderate or intense sound exposure. These phenomena suggest that marginal cells can behave heterogeneously, in terms of aminoglycoside uptake, after acoustic overstimulation. These drug-loaded marginal cells appeared to be exclusively localized over strial capillaries, and imply increased permeability of the strial blood-labyrinth barrier (BLB) that enabled enhanced drug trafficking into marginal cells. This sound-enhanced strial permeability (or micro-leakage) is not restricted to aminoglycosides, and has also been observed for other fluorescently-labeled proteins [38]. Regardless, once in marginal cells, which have a +10 mV potential greater than endolymph (+80 mV) [39], the cationic aminoglycosides would passively flow down the electrochemical gradient into endolymph and subsequently into hair cells (−70 mV) to exert its ototoxic effect [26]. Thus, the effect of acoustic trauma can be similar to loop diuretics, which also increase aminoglycoside uptake by marginal cells and hair cells [40].

Prominent vasodilation was observed immediately after chronic sound exposure (Fig. 4). It is possible that only large variations in vessel diameter can be reliably identified after cardiac perfusion and fixation. Although fixation may induce variation in strial capillary lumenal diameter, this was not observed in control tissues. Increased capillary lumenal diameters following noise exposure and fixation have been reported previously [41] and by casting techniques [42]. Intriguingly, vasodilation is not a prerequisite of sound-enhanced trans-strial trafficking. Enhanced trans-strial trafficking was also observed after recovery from sound-induced transient vasodilation (Fig. 5). This likely implicates a molecular mechanism is involved in enhanced trafficking of aminoglycosides.

TTS-enhanced trafficking of aminoglycosides across the strial BLB would presumably increase drug levels in endolymph, and it is this increased endolymph level that contributes to the observed increase in aminoglycoside uptake by hair cells. This effect would occur independently of sound stimulation itself since the open probability of the MET channel is hypothesized to remain relatively constant at rest or during stimulation [36]. Nonetheless, any spatio-temporal correlation of increased marginal cell loading of gentamicin, and uptake by hair cells in the proximity requires further study with better anatomical resolution.

C57Bl/6 mice are known for early onset of presbycusis after reaching three months of age [43]. Our subjects were 4–7 weeks of age, prior to the early onset of presbycusis in these mice, which were also used in prior murine studies using GTTR [3], [26], [28], [44]. Nonetheless, the stria vascularis and endolymphatic potentials from other strains of mice, such as CBA, may be more vulnerable by acoustic trauma [45]. Determining whether these murine strains harbor increased permeability (or trafficking) of aminoglycosides in stria vascularis during sound exposure, and display more potent sound and drug synergism, requires further study. In addition, it is unclear why of the large inter-animal variation on the effect of chronic sound exposure. A consistent mouse model harboring sound-induced (and/or age-induced) strial micro-ischemia will be advantageous for ototoxicity research, and provide opportunities to investigate any correlation between sound-induced strial micro-ischemia and enhanced drug uptake by hair cells. Our ultimate goal is to develop clinical strategies that maintain the integrity of the strial BLB and reduce aminoglycoside uptake by the cochlea during, or more frequently following sound-induced damage within the cochlea after blast or percussive noise exposure.

## Materials and Methods

### Conjugation and purification of GTTR

Gentamicin-Texas Red conjugate (GTTR) was produced as followed: 20% gentamicin (G-1264, Sigma, St. Louis, MO) in K_2_CO_3_ (100 mM, pH = 10) and 1% Texas Red (TR) succinimidyl esters (T6134, Invitrogen, Carlsbad, CA) in anhydrous N,N-dimethyl formamide were mixed in ratio of 212∶45 (v:v), and agitated together for one week at room temperature prior to purification. This protocol minimized the possibility of over-labeling individual gentamicin molecules with more than one TR molecule, and ensured the polycationic nature of the conjugate (GTTR). The reaction mixture was then diluted with 100 ml 5% Glacial acetic acid (GAA), and loaded onto pre-activated C-18 columns (Burdick and Jackson, Muskegon, MI) to purify the conjugate using reversed phase chromatography. Each C-18 column was activated by pre-rinsing with 5 ml MeOH and 10 ml 5% GAA. Unconjugated gentamicin was eluted using 100 ml 5% GAA; subsequently, unconjugated TR was eluted by rinsing with MeOH. To elute purified GTTR, each column was rinsed with 3 ml chloroform:MeOH:NH3 (30∶41∶30). GTTR solutions from multiple C-18 columns were pooled, aliquoted, lyophilized, and stored desiccated in the dark at −20°C. The amount of GTTR in each aliquot was calculated from the conjugation efficiency, and verified by determining the fluorescence intensity for an aliquot from each batch using a fluorimeter, and sample aliquots with known concentrations.

### Animal treatment, noise exposure and tissue preparation

Juvenile C57Bl/6 mice (4 to 7 weeks old) with positive Preyer's reflex were anesthetized with ketamine (65 mg/kg) and xylazine (13 mg/kg) intra-peritoneally (i.p.). Two mg/kg GTTR (gentamicin base in PBS, pH 7.4) or 200 mg/kg gentamicin in PBS, pH 7.4, was administered i.p. prior to, during or after sound exposure, depending on experimental design. The care and use of all animals reported in this study were approved by the Animal Care and Use Committee of Oregon Health & Science University (IACUC approval #IS00000351).

During prolonged or concurrent open field sound exposure, mice were housed in a custom-made cage with free access to food and water. The duration of sound exposure was either 3 or 6 hours at 100 dB SPL, or 18 hours over 3 days at 86 SPL. The cage was placed in the center of a sound proof chamber (Industrial Acoustic Company, New York, NY) with a total of 14 low, middle, and high frequency range loud speakers mounted on the slanted walls to achieve production of uniform sound fields with frequencies ranging from 20 Hz to 48 kHz. In-house developed software was used to flatten the speakers' acoustic response during production of limited and wide band noise (WBN) stimuli. A quarter-inch microphone (Brüel & Kjær, Denmark) located proximately above the animal housing, was capable of measuring the sound level in real time.

Closed sound delivery was only used for *acute* concurrent sound and drug exposure. Mice were anesthetized and systemically-administered with either GTTR or gentamicin at doses described above, at the beginning of 30-min concurrent sound exposure. A sound delivery tube was sealed into the left ear of anesthetized animals. An inner tube within the sound delivery tube was connected to a calibration microphone (ER-10B, Etymotic Research, Inc, Elk Grove Village, IL) for real time sound level monitoring. Sound level was fixed at 76 or 100 dB SPL. Only the left ears were exposed to an OBN centered at 12 kHz, while the right ears served as controls.

After sound exposure, deeply-anesthetized mice were promptly cardiac-perfused with PBS, then 4% formaldehyde. Cochleae from GTTR-treated mice were excised and post-fixed in 4% formaldehyde for one hour, then treated with Triton X-100 (0.5%) for 30 min. Gentamicin-treated cochleae were excised and post-fixed in 4% formaldehyde plus 0.5% Triton X-100 overnight, followed by immunolabeling (described below). All tissues were washed, counter-labeled with Alexa-488-conjugated phalloidin to assess hair bundle integrity, rinsed, and post-fixed with 4% formaldehyde [46]. GTTR or gentamicin (immuno)fluorescence in hair cells was documented from wholemounts of cochlear coils as described previously [26], [44].

Auditory brainstem responses (ABRs) to pure tones was used to ensure normal cochlear function prior to each experiment, and conducted as described earlier [47]. Briefly, each ear of anesthetized mice was stimulated individually with a closed tube sound delivery system sealed into the ear canal. The ABR to 1 ms rise-time tone burst stimuli at 8, 16 and 32 kHz, with 5 dB steps, was recorded and thresholds obtained for each ear. Only animals with normal bilateral ABR thresholds were used.

### Gentamicin immunofluorescence

Gentamicin-treated cochleae were immunoblocked in 10% goat serum in PBS for 30 minutes and then incubated with 5 µg/ml mouse monoclonal (Fitzgerald Industries, Concord, MA) gentamicin antisera for 2 hours. After washing with 1% goat serum in PBS, specimens were further incubated with 20 µg/ml Alexa-488-conjugated goat anti-mouse antisera (Molecular Probes, Eugene, OR) for one hour, washed three times, counter-labeled with Alexa-568-conjugated phalloidin, washed three times, and post-fixed with 4% formaldehyde for 15 minutes [46]. A subset of tissues were counter-labeled with 0.5 µg/ml DAPI nucleic acid stain (Molecular Probes, Eugene, OR) for 5 minutes prior to rinsing and post-fixation. For immunocytochemical controls, primary antiserum was omitted or replaced with gentamicin-adsorbed antisera and immunoprocessed as above.

### Imaging and data analysis

Specimens were whole-mounted in VectaShield (Vector Labs, Burlingame, CA) and observed using a Bio-Rad MRC 1024 ES laser scanning confocal system attached to a Nikon Eclipse TE300 inverted microscope. Fluorescent emissions were collected sequentially. For each set of experiments, all specimens in each group of experimental and control tissues were imaged at the same laser intensity and gain settings. Images from each experiment were identically prepared using Adobe Photoshop. Samples labeled with DAPI were imaged using an Olympus IX81 inverted microscope fitted with an Olympus Fluoview FV1000 (Japan) confocal laser microscope system.

For statistical analysis, GTTR or gentamicin (immuno)fluorescence intensity values in outer hair cell (OHC) cuticular plate region, from single optical sections were obtained by using the pixel histogram function (ImageJ, NIH), after removal of intercellular and extraneous tissue pixels using Photoshop [26], [48]. The cuticular plate region of OHCs had the highest level of GTTR fluorescence and was set as the region of interest (ROI) to be compared across experimental conditions (Figure S3). Two major regions along the murine basilar membrane of two and half turns were imaged. One was at the junction of the 1^st^ and the 2^nd^ turns, referred to “middle coil”, the other was towards to the end of 2^nd^ turn before reaching the more calcified hook region, referred to “basal coil”. Mann-Whitney nonparametric tests or paired t-tests, depending upon experimental design, were performed between sound-exposed and non-sound-exposed cochleae at each cochlear section to identify any statistically significant effect of sound on the fluorescent intensity of cellular drug uptake.

## Supporting Information

Figure S1 **Prior prolonged sound exposure increased GTTR uptake in OHCs.** GTTR (2 mg/kg) was injected i.p. during the final 30 minutes of an 18-hour WBN exposure over 3 days (6 hours per day, 86 dB SPL). The fluorescence intensity of the cuticular plate region of each OHC was scored from each confocal stack. Four stacks were imaged from each animal. Two sites were from the middle coil (circle) and 2 sites were from the basal coil (square). OHCs exhibited robust increase in GTTR fluorescence after prior prolonged sound exposure (n = 3), compared to control OHCs (n = 2). Horizontal lines depict the group mean of fluorescence intensity. Solid line: middle coil; dashed line: basal coil. The Mann-Whitney nonparametric test indicated that prolonged sound exposure exerted a statistically significant effect on GTTR fluorescence in OHC cuticular plates in both middle coil (p<0.001) and basal coil (p<0.05) locations compared to control cuticular plates.
(TIF)

Figure S2 **The dose-intensity relationship of GTTR or gentamicin (immuno)fluorescence.** The intensity of GTTR and monoclonal gentamicin (immuno)fluorescence is dose-dependent, unlike that for the polyclonal antibody. The panels on the right display the immunofluorescence emission of different gentamicin doses using monoclonal gentamicin antisera on Madin-Darby canine kidney (MDCK) cells. Scale bar  = 20 µm.
(TIF)

Figure S3 **Greater GTTR fluorescence in the cuticular plate region of OHCs.** **A**: Representative *xz* section image of organ of Corti from the basal cochlear turn of a GTTR-treated mouse. Prominent GTTR fluorescence (red) was observed in hair cells (OHCs, *; IHC, #), compared to surrounding supporting cells and structures. Green: phalloidin. SL: spiral limbus. TC: Tunnel of Corti. Scale bar  = 20 µm. **B**: Representative variation in GTTR fluorescence in the longitudinal axis of individual OHCs from the same cochlea, at various cochlear locations. Three to four OHCs per location were selected. More intense GTTR fluorescence occurred in the cuticular plate region (CP, gray area) regardless of cochlear location for individual OHCs. OHCs from more basal regions of the cochlea show the most intense GTTR fluorescence. HB: hair bundle.
(TIF)
