# Supplementary figures and images for: Acoustic Trauma Increases Cochlear and Hair Cell Uptake of Gentamicin
Source: PLoS One. 2011 Apr 28;6(4):e19130. doi: 10.1371/journal.pone.0019130 (PMC3084257; doi:10.1371/journal.pone.0019130)

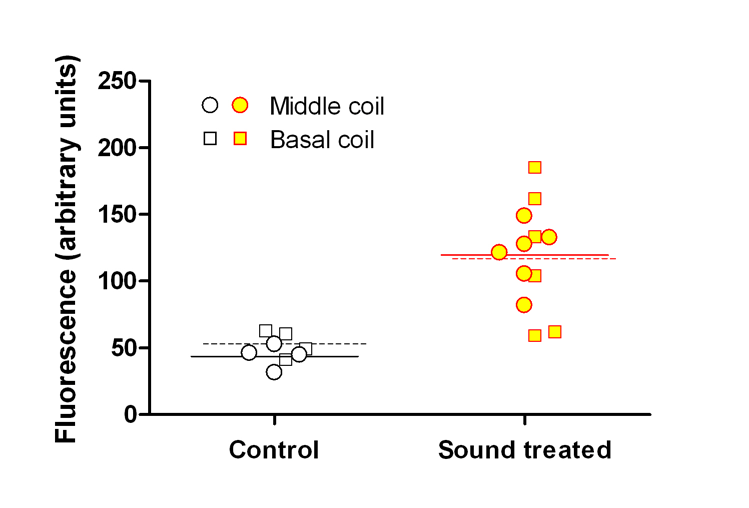

Supplement: Figure S1 — Prior prolonged sound exposure increased GTTR uptake in OHCs. GTTR (2 mg/kg) was injected i.p. during the final 30 minutes of an 18-hour WBN exposure over 3 days (6 hours per day, 86 dB SPL). The fluorescence intensity of the cuticular plate region of each OHC was scored from each confocal stack. Four stacks were imaged from each animal. Two sites were from the middle coil (circle) and 2 sites were from the basal coil (square). OHCs exhibited robust increase in GTTR fluorescence after prior prolonged sound exposure (n = 3), compared to control OHCs (n = 2). Horizontal lines depict the group mean of fluorescence intensity. Solid line: middle coil; dashed line: basal coil. The Mann-Whitney nonparametric test indicated that prolonged sound exposure exerted a statistically significant effect on GTTR fluorescence in OHC cuticular plates in both middle coil (p<0.001) and basal coil (p<0.05) locations compared to control cuticular plates. (TIF) [file pone.0019130.s001.tif]

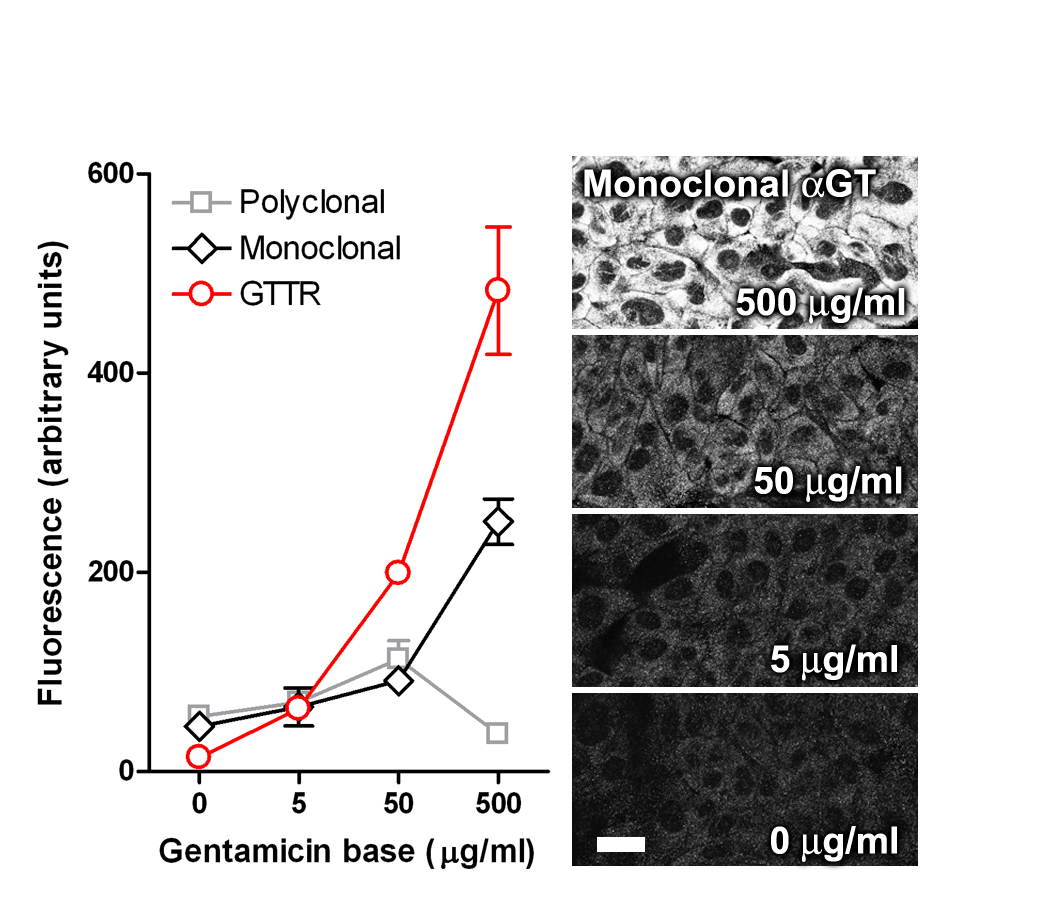

Supplement: Figure S2 — The dose-intensity relationship of GTTR or gentamicin (immuno)fluorescence. The intensity of GTTR and monoclonal gentamicin (immuno)fluorescence is dose-dependent, unlike that for the polyclonal antibody. The panels on the right display the immunofluorescence emission of different gentamicin doses using monoclonal gentamicin antisera on Madin-Darby canine kidney (MDCK) cells. Scale bar = 20 µm. (TIF) [file pone.0019130.s002.tif]

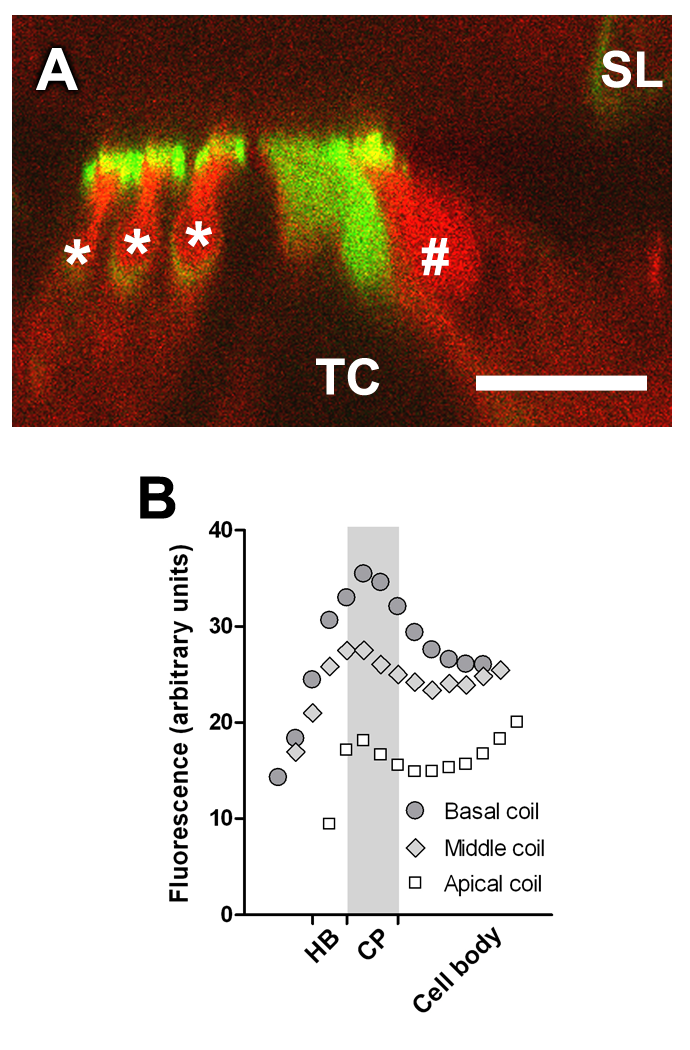

Supplement: Figure S3 — Greater GTTR fluorescence in the cuticular plate region of OHCs. A: Representative xz section image of organ of Corti from the basal cochlear turn of a GTTR-treated mouse. Prominent GTTR fluorescence (red) was observed in hair cells (OHCs, *; IHC, #), compared to surrounding supporting cells and structures. Green: phalloidin. SL: spiral limbus. TC: Tunnel of Corti. Scale bar = 20 µm. B: Representative variation in GTTR fluorescence in the longitudinal axis of individual OHCs from the same cochlea, at various cochlear locations. Three to four OHCs per location were selected. More intense GTTR fluorescence occurred in the cuticular plate region (CP, gray area) regardless of cochlear location for individual OHCs. OHCs from more basal regions of the cochlea show the most intense GTTR fluorescence. HB: hair bundle. (TIF) [file pone.0019130.s003.tif]
